# The Admission of Women to the Royal College of Surgeons

**Published:** 1908-07-18

**Authors:** 


### The Admission of Women to the Royal College of Surgeons.

The result of the poll of the Fellows and members of the Royal College of Surgeons of England on the question of the admission of women to the examinations of the College was announced last week. Of the Fellows, 1,043 voted out of a possible 1,373, and there is a majority of 288 in favour of the admission of women to the Membership and of 34 in favour of admitting them to the Fellowship; 8,543 Members voted out of about 13,800, and there is a majority of 703 against the admission of women to the Membership and of 1,216 against their admission to the Fellowship. Therefore over 63 per cent. of the Fellows voting, and nearly one-half of those qualified to vote, are in favour of admitting women to the membership, whilst of the Members 54 per cent. of those voting, and one-third of those qualified to vote, are opposed to their admission.

This difference in the opinion of the majority of the Fellows and of the Members is interesting; the adverse vote of the latter probably depends mainly on two causes—less personal knowledge of medical women and greater fear of their competition. There are probably thousands of practitioners who have never met a medical woman and who allow their imaginations to run riot in investing them with all kinds of dreadful attributes. All experience proves that wherever medical men are brought into personal contact with medical women they learn to respect them and are prepared to extend to them the right hand of fellowship. Fear of competition has doubtless been another factor in the result, but women have already so many portals available for qualification that the opening of the London Colleges would not be likely to induce many more to embark upon a medical career.

The Council of the College have deferred further consideration of the question until October, and their action then will be awaited with interest. After having passed a resolution to the effect that the admission of women to the examinations for the membership is desirable, they have, for the first time in the history of the College, consulted the whole body of the members, whilst stating that they would not necessarily be bound by the opinion of the majority. Will they accept the verdict of a comparatively small majority of the members voting, or will they adhere to their original decision, supported, as it is, by a majority of the Fellows and by the still more emphatically expressed opinion of the Fellows of the Royal College of Physicians? They may be influenced by the fact that considerably over one-third of the members did not even take the small amount of trouble required to mark and sign the reply postcards supplied to them, although the poll was kept open for several months to allow those living in distant parts to record their votes. As these non-voters certainly cannot be considered actively hostile, it follows that only one-third of the total number of members are definitely opposed to the admission of women.

The Council may possibly compromise the matter by admitting women to the Membership but not to the Fellowship, but such action would be illogical and unsatisfactory. It cannot be expected that women will be content to be regarded as an inferior order of practitioners, debarred from aspiring to the higher branches of the profession, and the Members would have a just grievance if a Council, elected by and composed exclusively of Fellows, were deliberately to exclude women from competing with those of their own order, whilst allowing them to compete on equal terms with the Members. The only satisfactory course is to follow the example of the College of Physicians and admit them to all the examinations. Women are now an integral part of the medical profession; it follows that all professional examinations, without distinction of place or status, should be thrown open to them. This must be done eventually. Why attempt to delay the removal of an obvious and indefensible injustice?

